# Spectroelectrochemistry of poly(3-hexylthiophenes) in solution

**DOI:** 10.1007/s11696-017-0277-6

**Published:** 2017-08-28

**Authors:** Kinga Kepska, Tomasz Jarosz, Anna Januszkiewicz-Kaleniak, Wojciech Domagala, Mieczyslaw Lapkowski, Agnieszka Stolarczyk

**Affiliations:** 1 Department of Physical Chemistry and Technology of Polymers, Silesian University of Technology, Strzody 9, 44-100 Gliwice, Poland; 2 Department of Inorganic, Analytical Chemistry and Electrochemistry, Silesian University of Technology, Bolesława Krzywoustego 6, 44-100 Gliwice, Poland; 3 Centre of Polymer and Carbon Materials, Polish Academy of Sciences, Curie-Sklodowskiej 34, 41-819 Zabrze, Poland

**Keywords:** Poly(3-hexylthiophene), Regioregularity, Electroprecipitation, Electrodeposition, Spectroelectrochemistry

## Abstract

**Abstract:**

The first comprehensive spectroelectrochemical account of the behaviour of regioregular (RR-P3HT) and statistical (ST-P3HT) poly(3-hexylthiophenes) in solution is presented, in contrast to the many reports dealing with P3HT films merely deposited from solution. The conducted experiments revealed that the two types of P3HTs behave in sharply different ways upon the application of electrochemical stimuli: ST-P3HT readily precipitates at mildly oxidative potentials, while the precipitation of the RR-P3HT takes place to a much lesser extent, even at higher potentials. The two polymers, studied via UV–Vis–NIR–EPR spectroelectrochemistry, exhibited properties mostly in line with earlier reports. Further study revealed that RR-P3HT largely remains in solution, even in its doped form, whereas only traces of the doped ST-P3HT are observed in solution in identical conditions. The high concentration of the doped RR-P3HT in solution can be explained by its ability to form soluble polymer agglomerates, in which the positive charge of the p-doped chains is stabilised by and delocalised over neighbouring, interacting undoped chains. These conclusions are consistent with SEM micrographs, which show that after cycling the potential of the electrode in a solution of ST-P3HT, a uniform layer is formed, covering most of the surface of the electrode, whereas in the case of RR-P3HT surface coverage is marginal and formed layer has the appearance of veined blotches.

**Graphical abstract:**

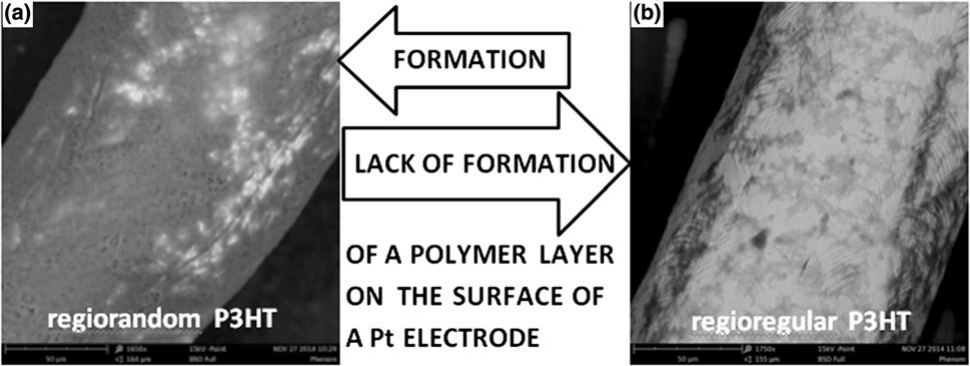

## Introduction

Poly(3-alkylthiophenes) (P3ATs) are  hole transporting conjugated polymers, which have found wide application in organic optoelectronics, due to their excellent properties and solution processability alike (Bento et al. 2012; Brinkmann 2011; Lee et al. 2013). These properties are shaped by the chemical structure of the repeat unit (Pal and Nandi 2003), the length of the polymer chain (Kline et al. 2005) and by its topology (Willot et al. 2013). Control over the structure of the repeat unit and the length of the polymer chain has been long exercised; achieving a desired topology of the polymer chain, however, requires the use of advanced polymerisation procedures (Dou et al. 2014; McCullough and Lowe 1992; Loewe et al. 1999; Osaka and McCullough 2008; Seung-Hoi and Rieke 2009) or the polymerisation of a regio-locked monomer (Jarosz et al. 2014; Zagorska and Krische 1990; Souto Maior et al. 1990).

Topology-controlled P3ATs, dubbed regioregular, have proven to significantly outperform their regiorandom analogues, with reports detailing a wide array of techniques being used for the study of both types of polymers (Deckers et al. 2015; Jiang et al. 2002). Existing works have largely focused on studying these systems in the solid state, leaving their properties of their solutions under-explored. This can be attributed to the fact that even soluble conjugated polymers are known to precipitate from solution upon doping, which hinders or outright forbids many types of investigations (Jacobs et al. 2016b).

Conversely, in the case of P3ATs, some reports of their doped-state solubility can be found upon scrutinising the available literature. (Dou et al. 2015; Jacobs et al. 2015, 2016a). This prompted our attempt to study the processes of their electrochemical precipitation/solubilisation as well as the associated doping/de-doping phenomena in solution. Here, we present the first comprehensive spectroelectrochemical account of the behaviour of regioregular (RR-P3HT) and regiorandom—statistical—(ST-P3HT) poly(3-hexylthiophenes) in solution, in contrast to the many reports dealing with P3HT films merely deposited from solution (Trznadel et al. 1998; Jiemsakul et al. 2017).

## Experimental

RR-P3HT and ST-P3HT have been prepared, with molecular weights of 10,000 and 48,000 g/mol, and dispersities of 1.3 and 5.1, respectively (as determined by size-exclusion chromatography, SEC).

### Materials

3-hexylthiophene (TCI, ≥98%), 2,5-dibromo-3-hexylthiophene (TCI, ≥97%), *t*-butylmagnesium chloride (Sigma-Aldrich, 2 M ether solution), dichloro-[1,3-bis(diphenylphosphino)propane]nickel(II) (Ni(dppp)Cl_2_) (Sigma-Aldrich, ≥97%) and anhydrous iron(III) chloride (Fisher Chemical, extra pure, SLR) were used as received, without further purification. Anhydrous tetrahydrofuran (ACROS Organics, 99.9%) was distilled over metallic sodium prior to use. All reactions were conducted under dry nitrogen or argon flow, in oven dried glassware.

### Synthesis of ST-P3HT via FeCl_3_ chemical oxidation method

The experimental procedure, based on the work of Niemi et al. (1992), was as follows: a dry 500 cm^3^ two-necked flask, equipped with a condenser, a gas capillary and a magnetic dipole, was charged with anhydrous iron(III) chloride (113 mmol) and chloroform (Sigma-Aldrich, >99%) (225 cm^3^) and purged with argon. Subsequently, 3-hexylthiophene (27.37 mmol) was added and the reaction was conducted for 1.5 h. Polymerisation was terminated by adding deionised water to the reaction mixture; the crude product was precipitated using acidified ethanol.

### Synthesis of RR-P3HT via GRIM method

Regioregular P3HT was synthesized via the McCullough GRIM method (Iovu et al. 2005), modified as per the work of De Girolamo (2007): a dry 100 cm^3^ three-necked flask, equipped with septum, a condenser, a gas capillary and a magnetic dipole, was purged with nitrogen and charged, via syringe, with 2,5-dibromo-3-hexylthiophene (6.13 mmol), anhydrous tetrahydrofuran (21.5 cm^3^) and *t*-butylmagnesium chloride (7.46 mmol). The reaction mixture was refluxed for 2 h, followed by addition of the Ni(dppp)Cl_2_ catalyst (0.0318 mmol) and heating for the next 1 h. The crude polymer was precipitated by quenching the reaction mixture in methanol.

### Purification of crude products

Obtained polymers were purified with sequential Soxhlet extraction with methanol (or ethanol, accordingly to the indicated precipitating solvent), hexane and chloroform. Vacuum dried chloroform fractions were characterized by means of ^1^H-NMR and SEC analyses and used as such in spectroelectrochemical investigations.

### Molecular characterisation of obtained products

^1^H-NMR analysis of both products was performed from solutions in CDCl_3_ on a Varian Unity Inova (USA) spectrometer with a resonance frequency of 300 MHz using TMS as internal standard. The number average molecular weights and dispersities were determined using a size-exclusion (SEC) chromatograph, equipped with an 1100 Agilent 1260 Infinity isocratic pump, an autosampler, a degasser, a thermostatic box for columns and a differential refractometric MDS RI Detector (USA). The molecular weight obtained by SEC was based on calibration with linear polystyrene standards (580–300,000 g/mol). Pre-column guard 5 μm (50 × 7.5 mm) and PLGel 5 μm MIXED-C (300 × 7.5 mm) column were used for separation. The measurements were carried out in THF (HPLC grade) as the solvent, at 30 °C with a flow rate of 0.8 cm^3^/min.

RR-P3HT: ^1^H-NMR (CDCl_3_, 300 MHz) δH, ppm: 6.98 (s, 1H), 2.81 (m, 2H), 1.76–1.66 (m, 2H), 1.47–1.34 (m, 6H), 0.91 (t, *J* = 6,9 Hz, 3H).

For ST-P3HT, ^1^H-NMR spectrum showed the same shifts, but with all peaks broadened, with no distinct multiplet structure. Moreover, the signal at 2.81 ppm is split into two: 2.81 and 2.57 ppm with relative integral ratios of 1.6–0.4.

### Spectroelectochemistry and voltammetric studies

The doping/dedoping processes occurring for the two types of P3HTs dissolved in chloroform were studied by coupled EPR–UV–Vis–NIR spectroelectrochemistry, whereas changes in the composition of the bulk solution were followed by fast, time-resolved UV–Vis spectroelectrochemistry.

Electrochemical investigations were performed using a standard three electrode cell, with a Pt wire working electrode, an Ag pseudo-reference electrode (calibrated vs. ferrocene/ferrocenium redox couple. All measurements were standardised so that the E_0_^FERROCENE^ corresponded to +0.42 V vs. Ag/Ag^+^) and a Pt mesh counter electrode. Consequently, the shown potentials vs. Ag/Ag^+^ are presented after being corrected to account for any shifts of the electrode potential. 0.1 M solution of tetrabutylammonium tetrafluoroborate (Sigma-Aldrich, >99.0%, electrochemical analysis grade) in chloroform was used as the electrolyte medium. Measurements were carried out on a Metrohm-Autolab PGSTAT100 N potentiostat (The Netherlands).

EPR–UV–Vis–NIR spectroelectrochemical measurements were performed using a tube cell (as shown in our earlier work: Jarosz et al. 2014) with a flat capillary section, fulfilling the role of a window for in situ UV–Vis–NIR measurements, equipped with an indium-tin oxide/quartz (Precision Glass & Optics, 20 ± 5 Ω/sq, 6 mm × 70 mm × 0.3 mm) (ITO) (for EPR–UV–Vis–NIR spectroelectrochemistry), an Ag pseudo-reference electrode and a Pt coil counter electrode. EPR spectra were acquired using a JEOL JES FA 200 X band spectrometer (Japan), with spin concentrations being calculated via double integration of the experimental spectrum. UV–Vis–NIR spectra were recorded using an Ocean Optics diode-array spectrometers set (QE65000 and NIRQuest 512) (USA).

**Fig. 1:**
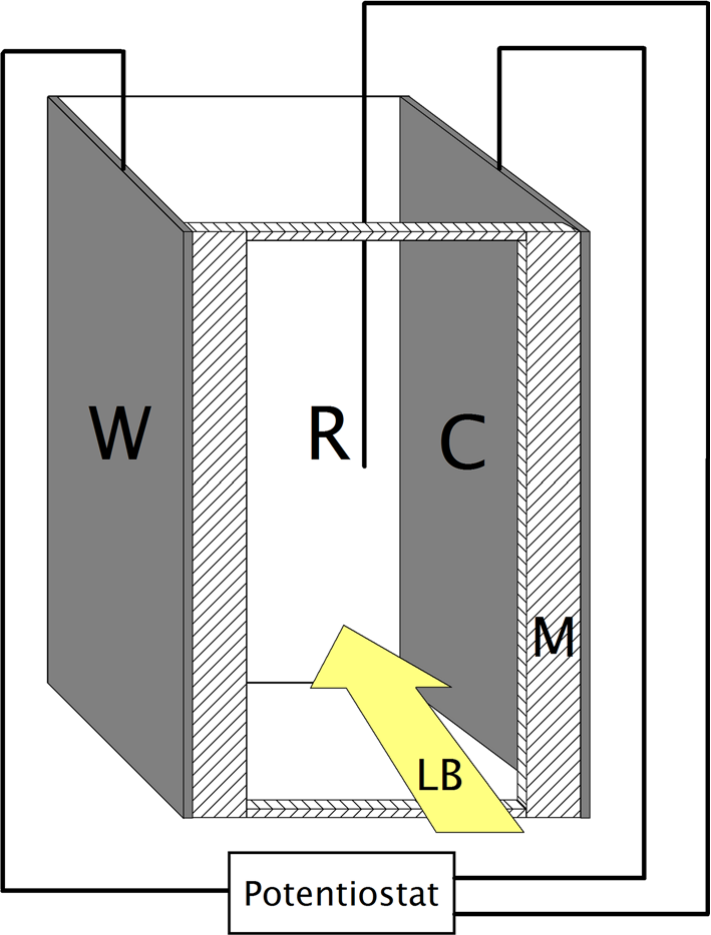
Scheme of the experimental setup for measuring the absorbance of the bulk solution. *W* working electrode, *C* counter electrode, *R* reference electrode, *M* PTFE mask, *LB* path of the light beam used for measurements

Fast, time-resolved UV–Vis spectroelectrochemical measurements of the absorption spectra of the bulk solution were conducted using the abovementioned Ocean Optics spectrometer set, using a 10 mm optical path length quartz cell as the experimental cell. This cell was equipped with an indium-tin oxide/quartz (Precision Glass & Optics, 20 ± 5 Ω/sq, 9 mm × 60 mm × 1 mm) working electrode, an Ag pseudo-reference electrode and an indium-tin oxide/quartz counter electrode. The working and counter electrodes were positioned parallel to each other, on the opposite sides of the experimental cell and the light beam was run through the solution between them. Any absorption signals related to the electrodes or near-electrode regions were eliminated through the use of a PTFE mask (as illustrated in Fig. 1) extending 1 mm towards the axis of the cell on each side. Spectral snapshots were taken every 0.5 s, which corresponds (at a potential scanning rate of 0.005 V/s) to a resolution of 0.0025 V, providing an extremely detailed picture of the changes in the composition of the experimental system induced by the potential applied to the working electrode. The surfaces of the formed P3HT layers were examined using a Phenom Pro X (Phenom World) scanning microscope (The Netherlands) with energy-dispersive detector (EDS).

## Results and discussion

**Fig. 2:**
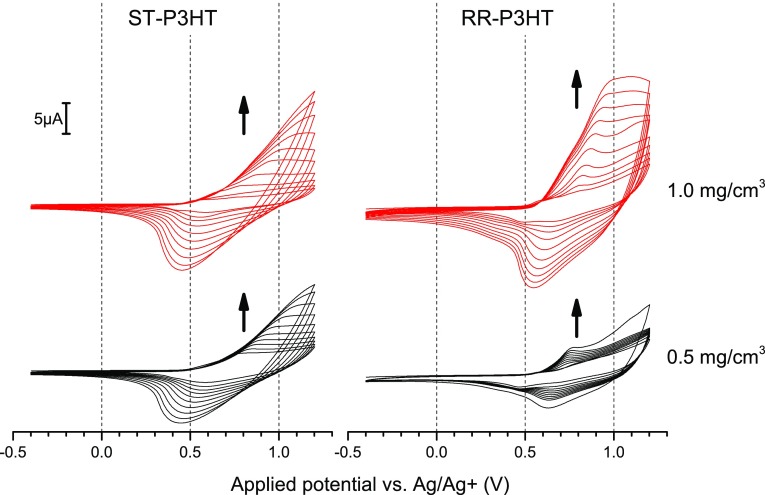
The electrochemical response of ST-P3HT (*left*) and RR-P3HT (*right*) of 1.0 mg/cm^3^ (*top*, *red*) and 0.5 mg/cm^3^ (*bottom*, *black*) solutions in 0.1 M TBATFB/chloroform solution. Potential scanning speed was 0.1 V/s

The first voltammetric cycle of the solutions of both RR-P3HT and ST-P3HT (Fig. 2) initially yields a response lacking any clear features. Repeated cycling leads to increasing the amount of oxidised P3HT present in the solution, resulting in precipitation on the electrode and the evolution of a redox response. In the case of RR-P3HT, the developing redox pair is located at less positive potentials than for ST-P3HT, evidencing better capability of accommodating charge carriers on the polymer chain.

**Fig. 3:**
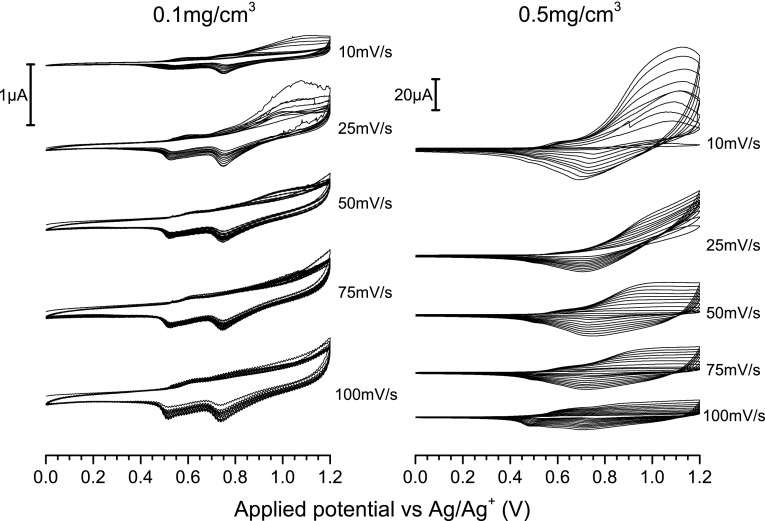
The electrochemical response of 0.1 mg/cm^3^ (*left*) and 0.5 mg/cm^3^ (*right*) RR-P3HT solutions in 0.1 M TBATFB/chloroform solution as a function of the applied potential scanning rate

The oxidation and reduction signals for both types of polymers are separated, indicating that doping/de-doping is tied with another process—in this case precipitation/solubilisation. Although reducing the concentration of the polymer solution from 1.0 to 0.5 mg/cm^3^ hinders the evolution of the redox signals, it does not significantly affect their shape, indicating that a similar equilibrium between the dissolved and precipitated polymer is achieved. Conversely, diluting the solution down to a concentration of 0.1 mg/cm^3^ (Fig. 3) yields a response with more pronounced individual current signals, which results from the fact that the deposition of the polymer becomes marginal, eliminating the overlap of current signals arising from the incipient polymer layer and the dissolved polymer in the near-electrode region. Interestingly, lowering the potential scanning rate appears to promote deposition, as seen in the case of a 0.5 mg/cm^3^ solution, where cycling the potential at a rate of 10 mV/s yields the largest cycle-by-cycle increases of the oxidation peak current.

**Fig. 4:**
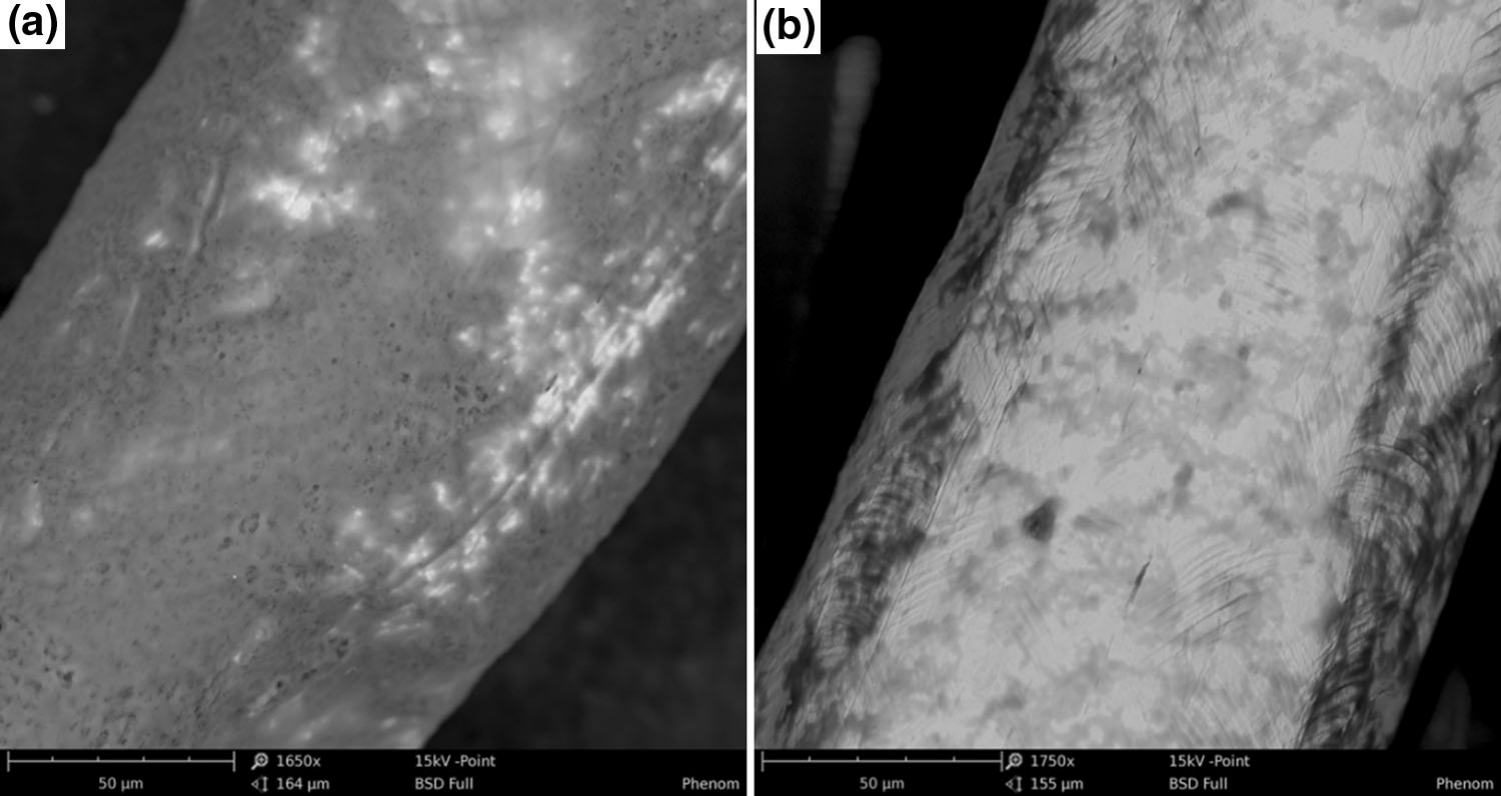
SEM micrographs of the surface of a platinum electrode following potential cycling in a polymer solution **a** ST-P3HT; **b** RR-P3HT. The *bright* and *dark areas* have been found to correspond to non-covered Pt and P3HT, respectively, using EDS and IR spectroscopic analysis

SEM photographs (Fig. 4) of the electrodes show that after cycling the potential of the electrode in a solution of ST-P3HT, a uniform layer is formed, covering most of the surface of the electrode. Conversely, sweeping the potential in the RR-P3HT solution, surface coverage is marginal and the formed layer has the appearance of veined blotches. The difference in film morphology is evidence that the ST-P3HT layer is significantly more amorphous than RR-P3HT, as expected on grounds of the existing literature reports (Liu et al. 2016; Tremel and Ludwigs 2014). Conversely, the poor surface coverage observed for RR-P3HT indicates that most of the electro-generated oxidised polymer remains in solution as no solid residue was left in the experimental cell. Consequently, in this work, we have put greater emphasis on the investigation of this unexpected behaviour of RR-P3HT.

**Fig. 5:**
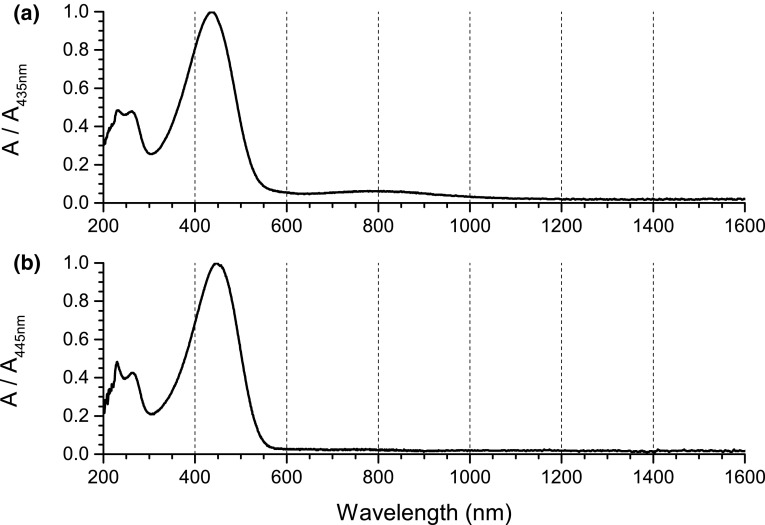
Normalised absorption spectra (absorbance of the undoped polymer peak, at 435 and 445 nm for ST-P3HT and RR-P3HT, respectively, assumed to be unity) of **a** ST-P3HT; **b** RR-P3HT. Spectra of the solution were taken prior to applying any electrochemical stimuli

**Fig. 6:**
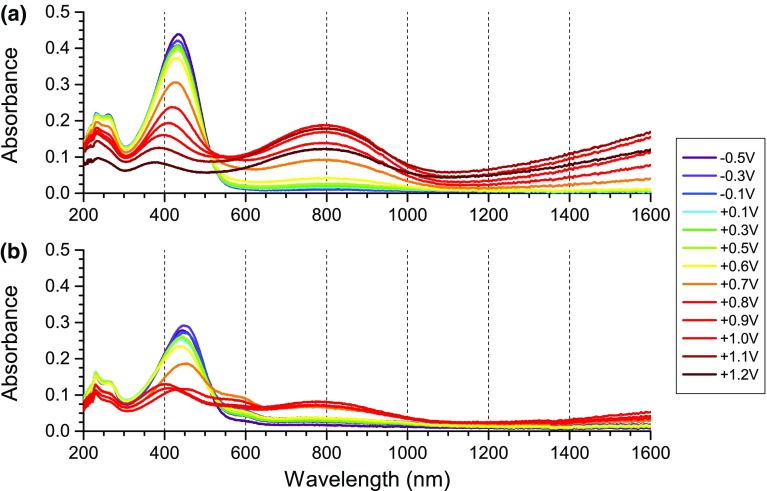
Absorption spectra of 0.25 mg/cm^3^ solutions of **a** ST-P3HT; **b** RR-P3HT in 0.1 M TBATFB/chloroform, plotted versus applied potential

UV–Vis–NIR spectra of the two polymers, registered prior to the application of electrochemical stimuli (Fig. 5), show that the ground state absorption maximum of RR-P3HT is located at lower energies than that of ST-P3HT, indicating a lesser band gap. In case of ST-P3HT, a weak band with a maximum at 800 nm reveals the presence of traces of the oxidised polymer—a trait brought on by the oxidative polymerisation procedure. Upon the application of increasingly oxidative potential to the solutions of both polymers, the ground state absorption peak diminishes, as an absorption signal, centred at 800 nm develops (Fig. 6), corresponding to the generation of charge carriers. In the case of RR-P3HT, however, the position of the ground state peak shifts significantly during this process, indicating changes in the geometry of the molecule and a shoulder signal at 560 nm can be observed.

When +1.2 V is applied to the solutions, both spectra become distorted, possibly due to precipitation of the oxidised polymer. This feature is more evident in the case of ST-P3HT, where the entire spectrum shifts downwards as the precipitate is eliminated from the optical path and settles at the bottom of the cell. The magnitudes of observed spectral changes differ between the two polymers, a feature brought on by the different effective conjugation lengths of the two polymers. Based on the noticeable elevation of the spectral baseline across all wavelengths, in the case of the regioregular polymer, a dispersive effect can also be postulated, possibly arising from the agglomeration of RR-P3HT chains due to π–π interactions.

**Fig. 7:**
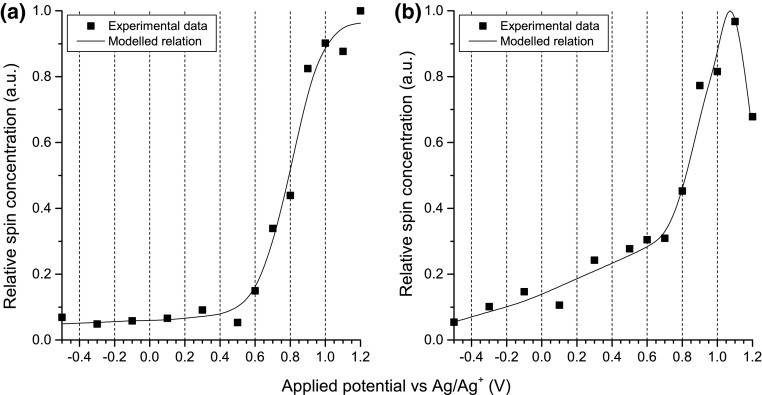
Relative spin concentrations plotted as a function of applied potential, registered for 0.25 mg/cm^3^ solutions of **a** ST-P3HT; **b** RR-P3HT in 0.1 M TBATFB/chloroform

The dependence of relative spin concentrations on applied potential for ST-P3HT (Fig. 7) exhibits an onset at +0.5 V, followed by a sharp increase and a plateau at high potentials. RR-P3HT in turn, shows an accelerating increase in the concentration of spin-bearing charge carriers starting from 0.0 V, and exhibits a maximum at higher potentials, indicating that the system reaches a doping level sufficient for recombination of spin-carrying polarons into spinless bipolarons to occur.

To study the precipitation and solubilisation of doped poly(3-hexylthiophenes), we have employed fast, time-resolved UV–Vis spectroelectrochemistry in a modified experimental setup (see Fig. 1).

**Fig. 8:**
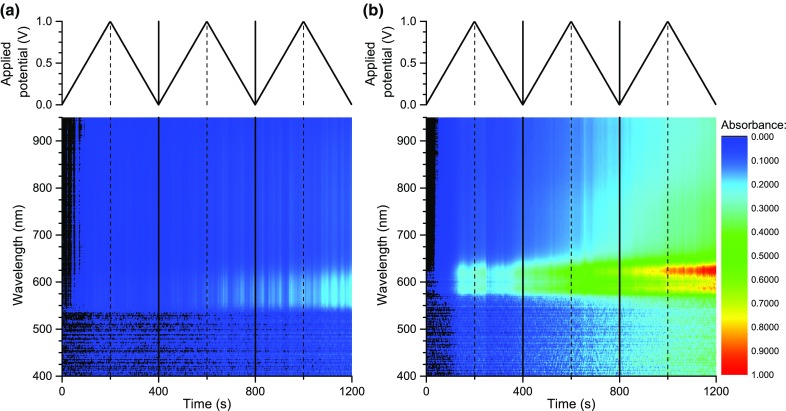
Time-resolved spectroelectrochemistry of bulk solution during potential cycling of 1.0 mg/cm^3^solutions of **a** ST-P3HT; **b** RR-P3HT in 0.1 M TBATFB/chloroform. Spectra are corrected for the absorption of the ground state polymer. The potential scanning rate used for these experiments was 0.005 V/s

In both cases, cycling the applied potential brings about evolution of absorption signals corresponding to the oxidised polymer. For RR-P3HT, however, absorbance higher by an order of magnitude is observed than for ST-P3HT (Fig. 7). The difference in molecular weight of the two types of polymers affects their diffusion coefficient, however, the time scale of the experiment was chosen so that diffusion equilibrium is achieved between periods where the potential applied to the working electrode is sufficiently high to oxidise the polymer. A difference in the magnitude of absorption changes was also observed for the two types of polymers via EPR–UV–Vis–NIR spectroelectrochemistry (Fig. 6), however, it can only partially account for this discrepancy. In light of the above evidence obtained by a number of techniques, we postulate that the observed difference in the absorption of the two types of polymers in this experiment is mainly due to superior solubility of RR-P3HT in the doped state (Fig. 8).

## Conclusions

In an attempt to study and compare the behaviour of regioregular and regiorandom poly(3-hexylthiophenes) in solution, multiple analytical techniques were engaged. The conducted experiments revealed a sharp discrepancy in the behaviour of the two types of P3HTs upon the application of electrochemical stimuli, with the regiorandom polymer readily precipitating at mildly oxidative potentials, while the precipitation of the regioregular polymer took place to a much lesser extent. The results of these electrodeposition processes were studied via SEM, revealing that after cycling the potential of the working electrode in a solution of ST-P3HT, a uniform layer is formed, covering most of the surface of the electrode, whereas only marginal surface coverage was achieved for RR-P3HT after potential cycling.

The two polymers were studied via UV–Vis–NIR–EPR spectroelectrochemistry, exhibiting properties typical for the two compounds and characteristic for their respective methods of synthesis. Further study of the observed difference in precipitation revealed that the regioregular polymer largely remains in solution, even in its doped form, whereas only traces of the doped regiorandom polymer are observed in solution in identical conditions. The presence of these traces can be attributed to fractions exhibiting low molecular weight and, consequently, higher solubility, based on their optical absorption maximum, which is expected due to the high dispersity of the synthesised regiorandom polymer. In turn, the high concentration of the doped regioregular polymer that was achieved in the reported experiments implies the existence of doped species exhibiting good solubility. The nature of this species is yet to be verified, but we can expect them to consist of p-doped P3HT chains interacting with numerous other undoped P3HT chains, “screening” the charged segments and preventing their precipitation. This interaction between doped and undoped polymer segments has already been reported by Zak et al. (2006), observing that a single electron hole can be delocalised over two oligomer molecules. We did not determine the solubility of the p-doped RR-P3HT; however, it being non-negligible was not expected in light of earlier reports and warrants further experimental exploration, as this feature appears promising in terms of processing the polymer for application purposes.
